# Multi-Input Speech Emotion Recognition Model Using Mel Spectrogram and GeMAPS

**DOI:** 10.3390/s23031743

**Published:** 2023-02-03

**Authors:** Itsuki Toyoshima, Yoshifumi Okada, Momoko Ishimaru, Ryunosuke Uchiyama, Mayu Tada

**Affiliations:** 1 Division of Information and Electronic Engineering, Muroran Institute of Technology, 27-1, Mizumoto-cho, Muroran 050-8585, Hokkaido, Japan; 2 College of Information and Systems, Muroran Institute of Technology, 27-1, Mizumoto-cho, Muroran 050-8585, Hokkaido, Japan

**Keywords:** multi-input deep neural network, speech emotion recognition, mel spectrogram, GeMAPS, focal loss function

## Abstract

The existing research on emotion recognition commonly uses mel spectrogram (MelSpec) and Geneva minimalistic acoustic parameter set (GeMAPS) as acoustic parameters to learn the audio features. MelSpec can represent the time-series variations of each frequency but cannot manage multiple types of audio features. On the other hand, GeMAPS can handle multiple audio features but fails to provide information on their time-series variations. Thus, this study proposes a speech emotion recognition model based on a multi-input deep neural network that simultaneously learns these two audio features. The proposed model comprises three parts, specifically, for learning MelSpec in image format, learning GeMAPS in vector format, and integrating them to predict the emotion. Additionally, a focal loss function is introduced to address the imbalanced data problem among the emotion classes. The results of the recognition experiments demonstrate weighted and unweighted accuracies of 0.6657 and 0.6149, respectively, which are higher than or comparable to those of the existing state-of-the-art methods. Overall, the proposed model significantly improves the recognition accuracy of the emotion “happiness”, which has been difficult to identify in previous studies owing to limited data. Therefore, the proposed model can effectively recognize emotions from speech and can be applied for practical purposes with future development.

## 1. Introduction

In real life, emotions reflect an individual’s state of mind and vitally constitute the affective factors in interpersonal communication [1]. Automatic emotion recognition is expected to be an effective technology for effortless communication and forms a major research hotspot in the field of artificial intelligence. Emotions can be identified from various sources of information, such as speech (voice), utterance transcript, facial expressions, and brain waves [2,3,4,5,6,7,8,9,10]. Among these sources, speech can be used for emotion recognition in face-to-face mode as well as in remote communication via telephone or video calls. In general, the speech characteristics for emotion recognition remain similar across all languages, and therefore, the same recognition model can be used for multiple languages [11].

To date, several machine learning methods have been proposed for speech emotion recognition [2]. With the remarkable improvement in computer performance in recent years, deep-neural-network-based methods have garnered considerable research attention as they automatically extract the features from audio samples and deliver a higher recognition performance than alternative machine learning algorithms [2]. In the field of speech emotion recognition (SER), mel spectrogram (hereinafter referred to as MelSpec) and acoustic parameters have been widely used as audio features [12,13]. In principle, MelSpec is a spectrogram converted into a mel scale based on human auditory characteristics and is typically expressed as an image. Moreover, the set of acoustic parameters determines the speech characteristics and is typically expressed as a feature vector. These two types of audio features offer distinct advantages and disadvantages. MelSpec can express the time-series variations in each frequency but fails to simultaneously manage multiple types of audio features. Conversely, the acoustic parameter set can operate multiple types of audio features but cannot provide their time-series information.

This study proposes a new SER model that can simultaneously learn MelSpec and acoustic parameters to complement their advantages and reduce their disadvantages. In this study, we employ the Geneva minimalistic acoustic parameter set (GeMAPS) [14] as the acoustic parameters, containing 88 parameters that are effective for SER. The proposed model is a multi-input deep learning model formed of three networks, specifically, for processing MelSpec in the image format, processing GeMAPS in the vector format, and integrating the output from these two networks to predict emotion. In this study, four types of emotion, namely, “anger”, “happiness”, “sadness”, and “neutrality”, are recognized using an emotional speech database, IEMOCAP [15]. In existing studies, the IEMOCAP database has been widely applied for SER [13], but owing to limited data, the accurate identification of the “happiness” emotion remains challenging [16]. In this study, we introduce a focal loss function [17,18] as the loss function of the proposed model to improve the recognition accuracy of such minority and difficult-to-identify emotions. The focal loss function can reduce the weight of easily classifiable data and focus more on data that are difficult to classify. We expect that the focal loss function will enable efficient learning of the minority and difficult-to-recognize emotion classes.

The remainder of this paper is organized as follows. The related work to this study is introduced in Section 2. Next, the development of the proposed model is presented in Section 3. Thereafter, the experimental methodologies and results are presented in Section 4 and Section 5, respectively. The present findings are discussed in detail in Section 6. Finally, the conclusions of this study, including the potential future scope of research, are summarized in Section 7.

## 2. Related Works

### 2.1. Emotional Speech Database

Emotional speech databases have been widely used to collect training data in the SER model and are broadly categorized into two types, i.e., actor-based databases [15,19] and natural speech emotional databases [20,21,22,23]. The actor-based databases use acting emotional speech data of trained actors, and the natural speech emotional databases store actual emotional speech data expressed naturally. The natural speech emotional databases enable us to construct SER models reflecting real emotions but have the limitations related to ethical issues. In contrast, the actor-based databases have been used in many SER studies because they are not subject to such limitations.

### 2.2. Audio Features Used in SER

Extraction of audio features is one of the important parts in SER. Audio features are categorized into two types: spectrograms and acoustic parameters. A spectrogram is a visual representation of the time variation of frequency spectrums and is typically provided in image format. Acoustic parameters are physical quantities representing voice characteristics such as F0, intensity, and frequency, and are typically represented with a vector format.

### 2.3. Models Used in SER

Models used in SER are classified into two types: traditional machine learning (ML) approaches and deep learning (DL) approaches. Commonly used traditional ML approaches are support vector machine [24,25], the hidden Markov model [26], the Gaussian mixture model [27], the K-nearest neighbor method [28,29], and decision tree [30]. Each of these traditional ML approaches has its own inherent advantages and disadvantages, but they share the commonality of requiring prior feature extraction from speech data [31]. In contrast, DL approaches can automatically learn speech characteristics without prior feature extraction and in most cases present higher recognition accuracy than traditional ML approaches. Current major DL approaches in SER are the dense neural network (DNN) [32,33], the convolutional neural network (CNN) [34,35], the recurrent neural network (RNN) [36], and long short-term memory (LSTM) [37]. DNN and CNN have been used to learn acoustic parameters and spectrogram images, respectively [2,13,38,39]. RNN and LSTM have typically been used to learn time-series variation of acoustic parameters [2]. Combined approaches of CNN and LSTM also have widely been employed [2,13,40]. Most of them generated feature vectors from spectrogram images by CNN and learn their time-series changes by LSTM. Moreover, there are CNN or LSTM-based models with attention structure [2,13,16,41].

Furthermore, there are deep-learning-based multi-input models that allow simultaneous learning of different audio features. Yenigalla et al. [42] proposed a multi-input CNN model that took a phoneme sequence and a spectrogram as input. Yao et al. [43] proposed a confidence-based fusion method for SER, which trained 32-dimensional vectors with RNN, 16 × 40-dimensional MelSpecs with CNN, and 384-dimensional vectors with DNN, separately. These existing multi-input models are similar to our model in that they took different forms of audio features as inputs. However, our study differs from theirs in two respects, namely using GeMAPS, a set of acoustic parameters effective for SER, and addressing the imbalanced data problem with the FL function for multi-class emotion recognition.

## 3. Materials and Methods

### 3.1. Dataset

The model construction and emotion recognition experiments were conducted using the speech data from the IEMOCAP database [15,44], which is provided by the signal analysis and interpretation laboratory at the University of Southern California [44] and is widely used in the field of emotion recognition research. The speech data contain 7 types of emotional voices performed in English by 10 actors (5 males and 5 females), segmented into improvised as well as scripted performances. Here, we employed the improvised speech data performed with four emotions, i.e., “anger”, “happiness”, “sadness”, and “neutrality”, to match the experimental conditions with previous studies [16,30,39,40,41] and to compare their results with ours.

The speech data can be received by e-mail by requesting the administrator from the IEMOCAP website [44]. The number of speech data was 1030 for “anger”, 594 for “happiness”, 1132 for “sadness”, and 1677 for “neutrality”, respectively. The speech data for each emotion were provided in WAV format. The emotion labels in the speech data can be identified by their time intervals described in the included text file. The total length of the speech data was approximately 12 h [41]. The longest and shortest speech lengths were 34.1 and 0.6 s, respectively, and the average speech length was 4.46 s. As explained in the next section, MelSpec and GeMAPS are extracted using the utterance sections assigned emotion labels in the speech data.

Emotion can be regarded as part of personal information; hence, it is important to consider the privacy of individuals involved in speech data. However, the speech data in the IEMOCAP database does not reflect the actual emotions of the actors due to their acting voices under a predefined experimental setting. Therefore, there are no ethical issues regarding the use of this speech data and its processed data.

### 3.2. Extraction of MelSpec and GeMAPS from Speech Data

MelSpec is a spectrogram calculated by replacing the frequencies with a mel scale, which is represented as image data displaying the temporal variations of amplitudes at each frequency. Here, the mel scale is a scale reflecting human speech perception with a frequency axis that plots lower frequencies in narrower intervals and higher frequencies in wider intervals. Recently, MelSpec has been used as an effective feature in machine-learning-based SER [45]. GeMAPS is a set of standardized acoustic parameters that is used to assess audio features for emotional speech analysis, represented as an 88-dimentional feature vector with 88 acoustic parameters related to frequency domain, energy/amplitude domain, and spectral balance [14]. GeMAPS has garnered tremendous attention as an effective audio feature in recent research on SER.

MelSpec and GeMAPS were extracted from the utterance section of the speech data of the IEMOCAP database according to the following procedure. First, the audio samples were sourced from the utterance section with a window size of 3000 ms and an overlap of 750 ms. In total, we acquired 735 samples for “anger”, 672 samples for “happiness”, 2010 samples for “sadness”, and 2333 samples for “neutrality”. Subsequently, MelSpec and GeMAPS were extracted from the acquired audio samples for each emotion. MelSpec was extracted using librosa [46], a Python package for music and audio analysis, where the number of frequency bins and time points were set to 128 and 376, respectively. GeMAPS was extracted as an 88-dimensional feature vector using OpenSmile [47], a standard audio feature extraction tool.

### 3.3. Model Construction and Emotion Recognition

**Figure 1:**
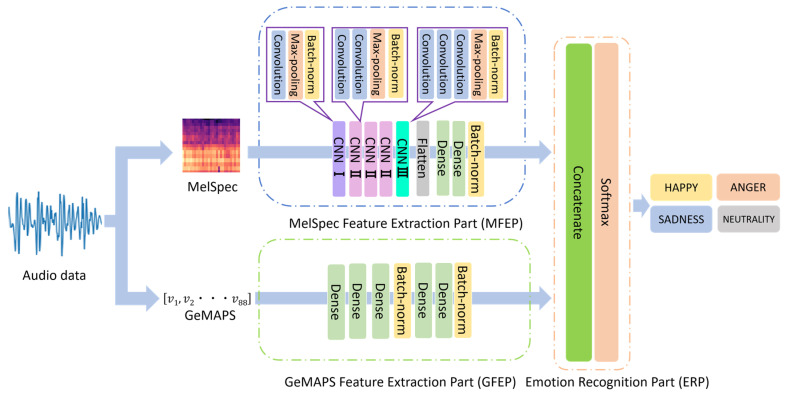
Architecture of proposed model.

The architecture of the proposed model is illustrated in Figure 1. The proposed model comprised a MelSpec feature extraction part (MFEP) based on a CNN [34,35], a GeMAPS feature extraction part (GFEP) based on a DNN [32,33], and an emotion recognition part (ERP). 

#### 3.3.1. Training Procedure of the Model

The training procedure of the model was as follows. The input to MFEP was a MelSpec in the form of a spectrogram image of 128 × 376 pixels. The feature extraction from the MelSpec was performed through the convolution layers, pooling layers, batch-normalization layers, a flatten layer, and the dense layers. The output from the MFEP was a 512-dimensional feature vector. The input to GFEP was the 88-dimensional feature vector of GeMAPS. Subsequently, the feature extraction from the GeMAPS was performed through the dense layers and batch-normalization layers. Similar to MFEP, the output from the GFEP was a 512-dimensional feature vector. In the ERP, these two feature vectors were simply concatenated without any special processing. The resulting 1024-dimensional feature was fed into the dense layers and subsequently transformed into a probability distribution for each emotion class using the softmax function [48]. The weight parameters were updated using the backpropagation algorithm [49]. In particular, Adam [50] was employed as the optimization function, whereas the ReLU function for MFEP and GEFP and the softmax function for ERP were used as the activation function. 

MFEP included one CNN I, three CNN II, one CNN III, and two dense layers. The filters in the max-pooling layers for CNN I, CNN II, and CNN III were set to a size of 2 × 2 with a stride width of 2. The filters in the convolution layers for CNN I, CNN II, and CNN III were set to the size of 3 × 3 with a stride width of 1. The number of filters was set to 64 for CNN I, 64, 128, and 256 for the three layers of CNN II, and 512 for CNN III. The number of neurons in the two dense layers after the flatten layer was set to 512. In total, the GEFP included 5 dense layers with 88, 128, 256, 512, and 512 neurons, respectively.

#### 3.3.2. Loss Functions Used in the Model

In this study, two types of loss functions were used, namely categorical cross-entropy loss (CCE) and focal loss (FL). CCE is a loss function that has been widely used in multiple emotion recognition methods based on deep learning, calculated as follows:

(1) $$CCE=-\sum_{k=1}^{N}t_{k}\cdot logy_{k}$$

where *N* denotes the number of emotion classes, $t_{k}$ indicates the ground truth for the *k*-th emotion class, and $y_{k}$ indicates the predicted probability for *k*-th emotion class. 

The FL function is a loss function proposed by Lin et al. [17]. In this study, to mitigate data imbalance among multiple classes, the following FL function was employed according to Yeung et al. [18]:

(2) $$FL=\sum_{k=1}^{N}{\alpha (1-y_{k})}^{\gamma }\cdot CCE$$

where $y_{k}$ denotes the predicted probability for the *k*-th emotion class, and α and γ indicate the hyper parameters set to 0.5 and 4.0, respectively.

#### 3.3.3. Emotion Recognition by the Model

The emotion recognition by the proposed model proceeded as follows. First, MelSpec and GeMAPS were extracted from an audio sample to be recognized and input into MFEP and GFEP, respectively. Subsequently, the two feature vectors obtained as outputs from the MFEP and GFEP were concatenated, and a probability distribution for each emotion class was generated through ERP. Finally, emotion recognition was performed by classifying the input audio sample into the emotion class with the highest probability.

## 4. Experiments

In this study, the audio samples obtained from eight actors (four males and four females) were used as training data, and those of the remaining two actors (one male and one female) were used as test data. In the emotion recognition experiments, we conducted the five-fold cross-validation test by swapping the male–female pair used as the test data. The recognition accuracy of the model was evaluated based on weighted accuracy (WA) and unweighted accuracy (UA), expressed as follows:

(3) $$WA=\frac{Number\;of\;correctly-classified\;audio\;samples}{Number\;of\;the\;whole\;test\;audio\;samples}$$

(4) $$UA=\frac{1}{K}\sum_{i=1}^{K}\frac{Number\;of\;correctly-classified\;emotion\;i}{Number\;of\;the\;whole\;test\;audio\;samples\;for\;emotion\;i}$$

In the experiments, to investigate the effect of using MelSpec and GeMAPS in combination (hereafter referred to as MelSpec+GeMAPS), we compared the recognition accuracy of the proposed model with those obtained using MelSpec or GeMAPS separately. Furthermore, to investigate the effect of the FL function, which was introduced to address the imbalanced data problem, we compared the recognition accuracies in case of using the CCE and FL functions as loss functions, hereafter referred to as MelSpec+GeMAPS (CCE) and MelSpec+GeMAPS (FL), respectively.

## 5. Results

### 5.1. Recognition Results of the Model

**Figure 2:**
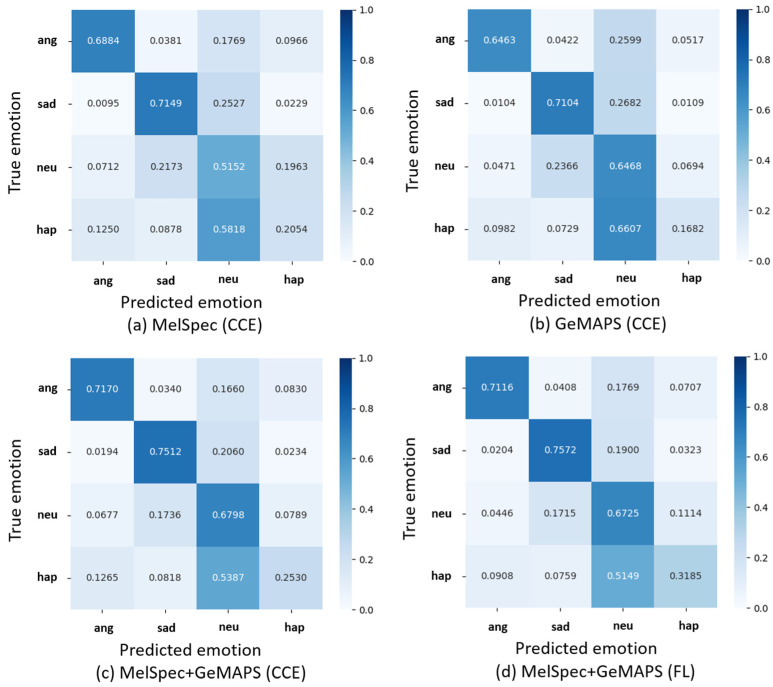
Confusion matrixes obtained in four experimental settings.

The overall recognition accuracy for all emotions are listed in Table 1. In addition, the confusion matrixes obtained in the four comparison experiments (Section 4) are illustrated in Figure 2, where the rate of the number of predicted emotions to that of true emotions is presented.

As observed from Table 1 and Figure 2, the combined use of MelSpec and GeMAPS significantly improved the recognition accuracy compared to the individual use of MelSpec and GeMAPS. These results indicated that the approach of combining MelSpec and GeMAPS was effective for SER. 

As indicated in Table 1, the FL function yielded a higher recognition accuracy than the CCE function in MelSpec+GeMAPS. Moreover, the comparison between Figure 2c,d implied an improvement of ~6% in the “happiness” class, which possessed the least training data. These results signified that the FL function could effectively solve the imbalanced data problem.

### 5.2. Comparison of Recognition Accuracy with Existing Methods

The recognition accuracies of the proposed model and those of existing methods are comparatively presented in Table 2, where “Proposed model” indicates MelSpec+GeMAPS (FL). As the existing methods, we selected the methods conducted under the speaker-independent experiments using the improvised acting speech of the IEMOCAP database, similar to our study. As the method proposed by Zhang et al. [39] is a multimodal model, only the SER result is provided for comparison with the proposed model. 

As discussed in Section 2, there existed the previous works on multi-input models, but no studies were conducted in the same experimental setting as our study. However, Yao et al.’s study [43] had the same experimental settings as our study, except that they conducted emotion recognition tests using both improvised and scripted data of the IEMOCAP database. Hence, in Table 2, we present their recognition accuracy for reference. However, Yenigalla et al. [42] did not specify in their paper whether a speaker-independent test was conducted; hence, their results are not shown in Table 2. 

Lee et al. [30] applied a hierarchical binary decision tree to 384-dimensional acoustic parameters and obtained WA and UA of 0.5638 and 0.5846, respectively. Neuman et al. [41] applied a CNN with attention structure to MelSpec and achieved WA of 0.6195 and UA of 0.6211. Satt et al. [40] applied a combined convolution-LSTM model to MelSpec and obtained WA of 0.6880 and UA of 0.5940. Yao et al. [43] applied a multi-input model based on DNN, CNN, and RNN to 384-dimensional acoustic parameters, MelSpec, and 32-dimensional acoustic parameters, respectively, and obtained WA of 0.5710 and UA of 0.5830. Li et al. [16] applied BLSTM-DSA, which introduced an LSTM with attention structure to 64-dimensional acoustic parameters and obtained WA of 0.6216 and UA of 0.5521. In contrast, this study applied a CNN and DNN-based multi-input model introducing the FL function to MelSpec and GeMAPS and achieved WA of 0.6657 and UA of 0.6149. From the above, it can be seen that the recognition accuracy of the proposed model was higher than or comparable to those of the existing methods, including the state-of-the-art methods.

## 6. Discussion

As shown in Section 5, in the existing methods, both Neumann et al. [41] and Satt et al. [40] used MelSpec as audio features. Compared to the method by Neumann et al., the proposed model was slightly inferior in UA but greatly superior in WA. Compared to the result of Satt et al., the proposed model showed recognition accuracy comparable to theirs. Lee et al. [30], Zhang et al. [39], and Li et al. [16] used acoustic parameters as audio features. Remarkably, the proposed method achieved significantly higher accuracy than all of these methods. Yao et al. [43] used both MelSpec and acoustic parameters based on a multi-input model. Both WA and UA of the proposed method considerably outperformed those of Yao et al.’s model. 

From the above, we can say that the recognition accuracy of the proposed model was higher than or comparable to that of existing methods, which could be attributed to two possible reasons. First, the approach of combining MelSpec and GeMAPS in the proposed model is novel because in the existing methods, MelSpec and GeMAPS have been used separately with various models. As discussed earlier, the spectrogram can represent the time-series variations in each frequency band but fails to simultaneously manage various audio features. In contrast, the acoustic parameter set can simultaneously handle multiple audio features but cannot extract their time-series variations. Accordingly, the proposed model enabled complementary learning of the respective merits of MelSpec and GeMAPS. As presented in Table 1 and Figure 2, the proposed model achieved a significant improvement in the recognition accuracy compared to separately using MelSpec and GeMAPS. Second, the FL function contributed significantly to the imbalanced data problem. In the latest study [16], the recognition accuracy of the “happiness” emotion was only 15.13% because identifying “happiness” from “neutrality” is challenging, especially with a limited extent of training data. In contrast, the proposed model succeeded in improving the recognition accuracy of “happiness” to 31.85%. The FL function penalized the easily identifiable classes, thereby promoting the learning of classes that are difficult to identify. Therefore, it effectively recognized the minority and difficult-to-identify classes. We conjecture that the FL function enhanced the learning of “happiness”, which is a minority and difficult-to-recognize emotion class, and improved its recognition accuracy.

The proposed model poses three major limitations for practical applications. The first limitation is to require improvement of the quality and quantity of the training data. As the proposed model was developed using the speech data performed by 10 actors, the emotion labels may not necessarily correspond with the actors’ own emotions. To realize more accurate emotion recognition, extensive amounts of training data should be acquired based on the speaker’s actual emotions. For that purpose, individual privacy needs to be considered. The second limitation is associated with the enhancement of the presentation method of the recognized emotions. Here, the proposed model predicted only one emotion for the input speech. However, individuals may experience multiple emotions at the same instant. Therefore, identification and presentation of all possible emotions is desirable for practical purposes. The third limitation pertains to the simplification of the model. As the proposed model is a deep neural network with numerous layers, it requires expansive computational resources to process the copious number of parameters. Nonetheless, the proposed model should function effortlessly on devices with limited hardware resources and on applications with strict latency requirements without any deterioration in recognition accuracy. To this end, model compression technology is essential [51,52].

## 7. Conclusions

MelSpec and GeMAPS have been widely used as effective audio features in SER. This study proposed a new SER model based on a multi-input deep neural network, which enabled simultaneous learning of both these audio features. The conclusions of this study are summarized as follows:

1. The proposed model delivered higher recognition accuracy than using MelSpec and GeMAPS separately.
2. The recognition accuracy of the proposed model was higher than or comparable to those of the state-of-the-art existing methods.
3. The introduction of the FL function improved the recognition accuracy of the “happiness” emotion.

In future, the quality and quantity of the training data should be enhanced, a method must be developed for presenting multiple emotions, and model compression should be performed to realize the practical applications of the proposed model. In addition, although this study employed the deep neural networks as the classification model, in future, we will compare among multi-input models using other classification models such as support vector machine [24,25] and random forest [53] and investigate the usefulness of combining MelSpec and GeMAPS. 

In SER, dealing with noise is a critical issue [13]. Most studies, including this study, have conducted evaluation experiments using clear speech recorded under favorable conditions. In reality, however, various types of noise can be introduced into speech. To apply the model to real speech data, it is necessary to introduce high-performance noise reduction [54] in the model itself or in preprocessing. 

Furthermore, emotions have an aspect of personal information. Speech data collected from individuals should be given the utmost ethical consideration not only in its actual use in emotion recognition systems but also in its management and operation. Researchers need to create technologies according to ethical guidelines.

## Figures and Tables

**Table 1:** Recognition accuracy for four experimental settings.

Features	WA	UA
MelSpec (CCE)	0.5710	0.5310
GeMAPS (CCE)	0.6130	0.5429
MelSpec+GeMAPS (CCE)	0.6597	0.6003
MelSpec+GeMAPS (FL)	0.6657	0.6149

**Table 2:** Comparison with state-of-the-art methods.

Author	Year	Features	Method	WA	UA
Lee et al. [30]	2011	Acoustic parameters	Hierarchical binary decision tree	0.5638	0.5846
Neumann et al. [41]	2017	MelSpec	Attention-CNN	0.6195	0.6211
Satt et al. [40]	2017	MelSpec	Convolution-LSTM	0.6880	0.5940
Zhang et al. [39]	2020	Acoustic parameters	DNN	0.6272	N/A
Yao et al. [43]	2020	Acoustic parameters, MelSpec	DNN+CNN+RNN multi-input model	0.5710	0.5830
Li et al. [16]	2021	Acoustic parameters	BLSTM-DSA	0.6216	0.5521
Proposed model	2023	MelSpec+GeMAPS	CNN+DNN	0.6657	0.6149

## Data Availability

The program code used in the research can be obtained from the corresponding author upon request. The data can be obtained upon request to the administrator of the web site [44].
